# Germline and reproductive tract effects intensify in male mice with successive generations of estrogenic exposure

**DOI:** 10.1371/journal.pgen.1006885

**Published:** 2017-07-20

**Authors:** Tegan S. Horan, Alyssa Marre, Terry Hassold, Crystal Lawson, Patricia A. Hunt

**Affiliations:** School of Molecular Biosciences, Center for Reproductive Biology, Washington State University, Pullman, Washington, United States of America; Cornell University, UNITED STATES

## Abstract

The hypothesis that developmental estrogenic exposure induces a constellation of male reproductive tract abnormalities is supported by experimental and human evidence. Experimental data also suggest that some induced effects persist in descendants of exposed males. These multi- and transgenerational effects are assumed to result from epigenetic changes to the germline, but few studies have directly analyzed germ cells. Typically, studies of transgenerational effects have involved exposing one generation and monitoring effects in subsequent unexposed generations. This approach, however, has limited human relevance, since both the number and volume of estrogenic contaminants has increased steadily over time, intensifying rather than reducing or eliminating exposure. Using an outbred CD-1 mouse model, and a sensitive and quantitative marker of germline development, meiotic recombination, we tested the effect of successive generations of exposure on the testis. We targeted the germline during a narrow, perinatal window using oral exposure to the synthetic estrogen, ethinyl estradiol. A complex three generation exposure protocol allowed us to compare the effects of individual, paternal, and grandpaternal (ancestral) exposure. Our data indicate that multiple generations of exposure not only exacerbate germ cell exposure effects, but also increase the incidence and severity of reproductive tract abnormalities. Taken together, our data suggest that male sensitivity to environmental estrogens is increased by successive generations of exposure.

## Author summary

Developmental exposure to manmade chemicals that interfere with endogenous hormones (endocrine disrupting chemicals) has been reported to adversely affect male reproductive health, increasing the incidence of reproductive tract abnormalities and reducing sperm production. Experimental evidence suggests that some exposure effects can persist in unexposed descendant males. To date, however, studies of these transgenerational effects have failed to accurately model human exposure, which spans multiple generations and involves an increasing number and diversity of endocrine disrupting chemicals. Using a quantitative measure of exposure effects on the germline, we assessed the effects of successive generations of estrogenic exposure in mice. We found that multiple generations of exposure not only exacerbated previously reported effects on the male germline, but elicited reproductive tract defects that increased in frequency and severity. These results have important implications for human reproductive health, suggesting that multiple generations of exposure to common endocrine disrupting chemicals may increase male sensitivity to exposure.

## Introduction

Data from human populations around the world provide evidence of a marked decline in male fertility during the past several decades. For example, a comprehensive analysis in 2000 of data from more than 100 studies in Western countries provided evidence of a decline in human spermatogenesis during the preceding 50 years [1]. More recent longitudinal cross-sectional studies suggest reductions in both sperm count and quality among young men (ages 18–37) in China (2001–2015;[2]), Spain (2001–2011;[3]), France (1989–2005;[4]), Denmark (1996–2010;[5]), and Finland (1998–2006; [6]). Changes in sperm production have coincided with increases in the incidence of other reproductive defects, including hypospadias, cryptorchidism, and testicular germ cell cancers (reviewed in [7]), and the combined spectrum of reproductive effects has been termed testicular dysgenesis syndrome (TDS; [8]). The observed changes correspond to the rapid introduction of manmade chemicals in the postwar era, and were originally hypothesized to result from exposure to maternally- or environmentally-derived estrogens [9]. Subsequent experimental data, however, have provided evidence that male reproductive abnormalities can be induced by developmental exposure to different types of endocrine disrupting chemicals (EDCs; reviewed in [10,11]). Given the rapid increase in the variety and ubiquity of EDCs in our environment and the adverse reproductive effects ascribed to some of these chemicals, the implications for humans are significant.

The most compelling evidence of an effect of developmental estrogenic exposure on human male reproductive health comes from studies of diethylstilbestrol (DES) exposed sons. From the 1940s through the 1970s, DES was prescribed to millions of pregnant women to prevent miscarriage. This treatment not only was not efficacious, but increased the incidence of a variety of reproductive disorders, including cancers in both male and female offspring (reviewed in [12]). Although DES daughters have been studied more extensively, in DES sons and in male mice exposed prenatally to DES, the incidence of cryptorchidism, underdeveloped testes, and testicular cancer is increased, and sperm count and quality is decreased [13–16]. Further, although the lack of information on sources, levels and timing of exposure precludes systematic studies of other developmental estrogenic exposures in humans, epidemiological studies suggest etiological links between environmental exposures and changes in spermatogenesis and the incidence of testicular germ cell cancers of fetal origin (reviewed in [7,17]).

Evidence that the effects of exposure may be transmitted to subsequent, unexposed generations is accumulating. Because exposure not only can directly affect the exposed individual (F0), but also his or her germline, effects evident in generations derived from this germline (the F1 in the case of male exposure, but both the F1 and F2 generations in the case of fetal exposure involving the female) are said to be multigenerational. For an effect to be considered transgenerational, it must be evident in the first unexposed generation (F2 and F3 for male and female exposures, respectively). Transgenerational effects in mammals–presumably resulting from epigenetic changes to the germline–have been reported in numerous studies (e.g., [18–25]). Few studies, however, have focused on germ cells [26–28], and the evidence supporting the persistence and transmission of specific germline alterations remains insufficient to convince some skeptics (e.g. [29–31]). Direct effects on the developing male germline have been induced by perinatal exposure to exogenous estrogens in mice and rats, with adverse effects reported on both gonocyte number and adult sperm production [32–36]. In addition, we previously demonstrated an effect on the developing spermatogonial stem cell (SSC) induced by brief postnatal exposure coinciding with the formation of the SSC lineage in male mice and evident as a reduction in meiotic recombination levels in descendant spermatocytes [37].

Documenting transgenerational effects in humans is challenging. Assessing potential transgenerational transmission of DES-induced effects will require analysis of an additional generation of descendants and, for most common environmental chemical contaminants, assessment likely will never be possible due to the nature of human exposure: Typically, humans are not exposed for only a single generation. Instead, exposures persist over time or become more diverse as new chemical variants are introduced.

**Fig 1:**
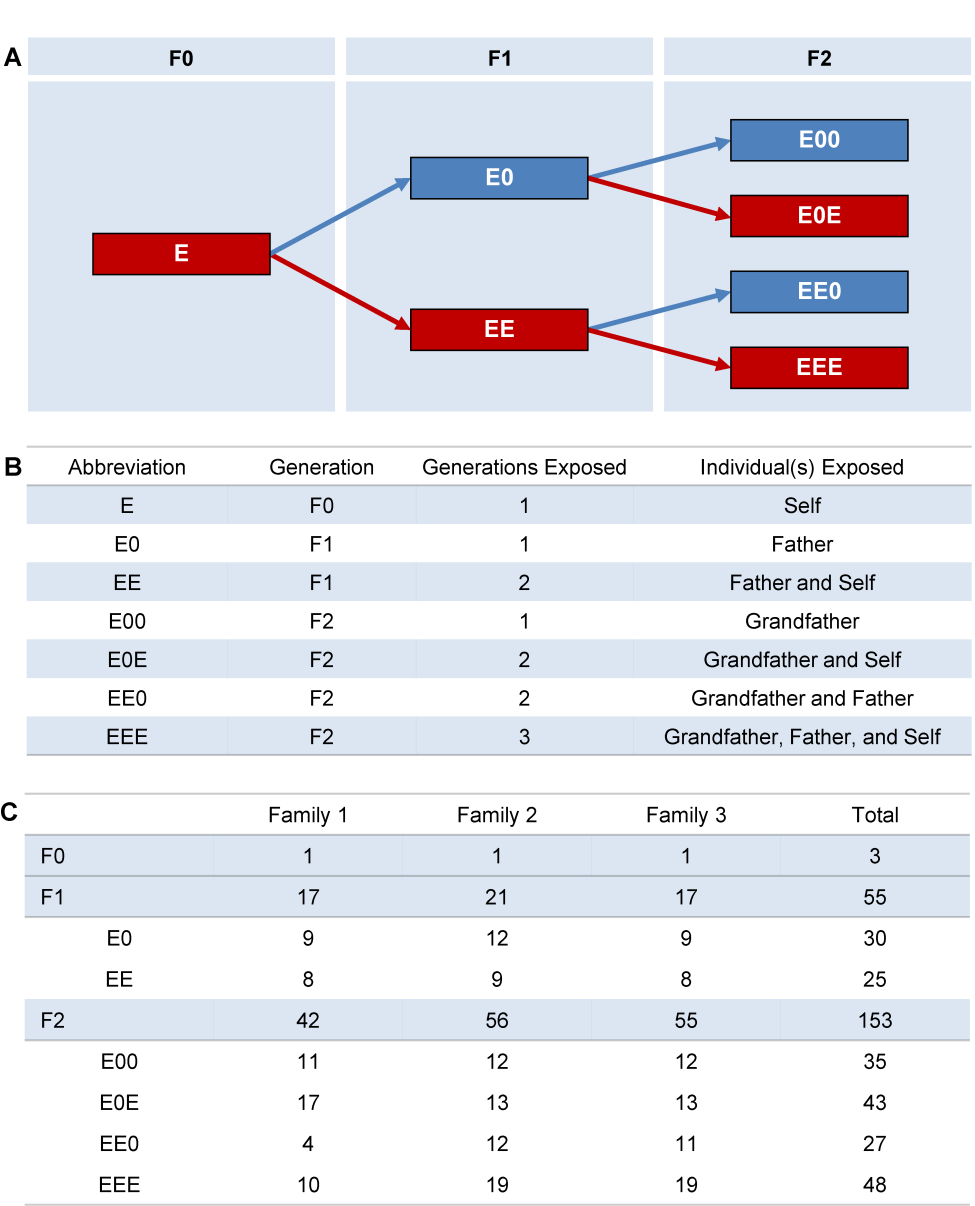
Multigenerational exposure paradigm. (A) F0 males (designated E) were treated from 1–12 dpp with 0.25 ng/g ethinyl estradiol and bred with unexposed females to produce F1 males that received daily oral doses of either ethinyl estradiol (EE; red) or placebo (E0; blue) from 1–12 dpp. Representative F1 males chosen at random were bred with unexposed females to generate F2 generation males that, like the F1, were either exposed (E0E and EEE; red) or placebo treated (E00 and EE0; blue). After mating, all males were killed at 12 weeks of age for reproductive tract and testis analysis (B) Summary of abbreviations and specific exposure(s) represented by each. (C) Summary of animal numbers in each treatment group.

To our knowledge, the effects of successive generations of exposure on male reproduction have not been addressed. Thus, we decided to use a sensitive, quantitative measurement of exposure, meiotic recombination, to assess the effect of exposures spanning multiple generations. We utilized an outbred mouse model and a complex three-generation scheme (Fig 1A) involving low-dose, neonatal exposure to the synthetic estrogen, ethinyl estradiol. Our data not only demonstrate an increase in the severity of exposure-induced effects on meiotic recombination with successive generations of exposure, but also an unexpected increase in both the incidence and severity of male reproductive tract aberrations. Taken together, our findings suggest that continued exposure spanning several generations will have cumulative effects on male reproductive health.

## Results

We recently reported that neonatal estrogenic exposure induces permanent meiotic effects in adult outbred CD-1 and inbred C3H, but not C57BL/6J male mice [37]. Germ cell transplantation experiments demonstrated that the meiotic phenotype was due to alterations in the spermatogonial stem cells (SSCs) of the testis, a lineage thought to be determined during the window of exposure used in the study [38–40]. The SSC is many cell divisions upstream of meiotic entry; thus, rather than affecting the meiotic DNA double strand break (DSB) repair process per se, it is likely that changes induced in the SSC population altered the recombination set point. Consistent with this, we found no difference in DSB formation or synaptonemal complex length in exposed and control males [37]. Studies to determine how exposure alters the SSC epigenome are in progress, and ultimately will provide important insight to recombination control in male mammals. In the interim, because recombination provides a quantitative measure of an exposure effect on the germline, it provides a direct means of tracing effects through generations to determine if they are multi- or transgenerational.

Our previous studies demonstrated that exposure to either bisphenol A (BPA) or ethinyl estradiol from 1–12 days postpartum (dpp) significantly reduced meiotic recombination (as assessed by the number of foci of the DNA mismatch repair protein, MLH1 in pachytene spermatocytes) in adult males. Daily oral doses of 0.25 ng/g ethinyl estradiol (roughly equivalent to a daily oral contraceptive dose) exerted the strongest effect, causing a 5% reduction in MLH1 values in inbred C3H males [37]. Although this difference appears subtle, the direct biological consequence is the elimination of spermatocytes. Cells with one or more pairs of homologous chromosomes that fail to form a crossover site will not yield sperm, because the presence of unpaired chromosomes at the first meiotic division triggers checkpoint-induced spermatocyte elimination [41,42].

We were interested not only in analyzing second- and third-generation descendants of exposed males for the transgenerational persistence of meiotic effects, but also in assessing the effects of successive generations of exposure. Accordingly, we developed the three-generation exposure protocol outlined in Fig 1 and S1 Fig, and conducted all analyses on 12-week-old adult males. To track both generational and individual exposure history, F0 founder exposed males were designated as ‘E’, and ‘E’s and ‘0’s used to designate exposure or placebo treatment, respectively in subsequent generations (Fig 1B and 1C). For example, EE males represent F1 generation exposed sons with two generations of exposure; E00 males, F2 grandsons two generations removed from the founder exposure; and EEE males, F2 grandsons with three successive generations of exposure. In this paradigm, E0 and E00 males serve as important negative controls for EE and EEE exposure groups.

To eliminate genetic variability, we initiated our three-generation studies using inbred C3H males; however, four of the nine founder males proved infertile with orchitis. When we attempted the study using inbred 129 males orchitis was not observed, but only one of four exposed males proved fertile. We next turned to outbred CD-1 males.

Although the use of outbred animals introduces genetic variability, our previous studies demonstrated meiotic effects in neonatally exposed CD-1 males (i.e., an average decrease in adult males of 1.3 MLH1 foci for BPA and 2.5 for ethinyl estradiol) and suggested that exposed CD-1 males are fertile [37]. The highly significant difference between ethinyl estradiol and placebo exposed males suggested that, despite genetic variation, we would be able to discern generational differences using CD-1 males, thus, we conducted our studies on this outbred background.

### Exposure-induced reproductive tract abnormalities are additive

**Fig 2:**
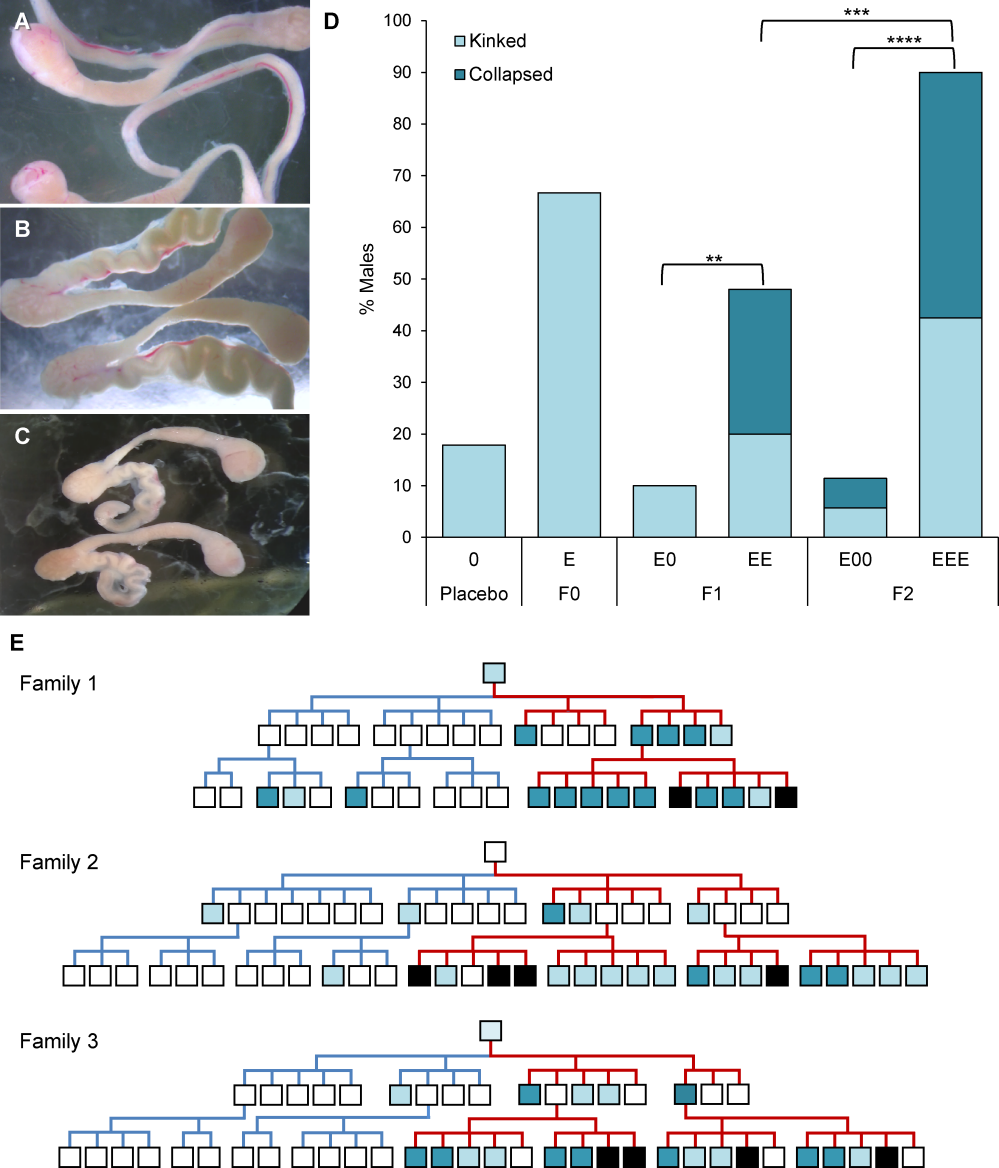
Successive generations of estrogenic exposure increase both incidence and severity of vas deferens malformations. (A-C) Epididymis and attached vas deferens showing normal (A), ‘kinked,’ characterized by a convolution along the length of the vas deferens (B), and ‘collapsed’, characterized by curling of the ‘kinked’ duct on itself (C) phenotypes. (D) Comparison of the frequency of kinked (light blue) or collapsed (dark blue) phenotypes in placebo controls (n = 27), the 3 F0 founders, 30 E0 and 25 EE F1 sons, and 35 E00 and 40 EEE F2 grandsons. Incidence of abnormal phenotypes was significantly higher after successive generations of exposure: Asterisks denote level of significance of comparisons (see text for details). (E) Pedigrees of exposed families with vas deferens phenotype denoted by square color: normal (white), kinked (light blue), and collapsed (dark blue), and black denoting fibrotic testes. Blue lines of descent indicate placebo treated lineages, red denote estrogen treated. For simplicity, E0E and EE0 F2 males have been excluded; see S1 Fig for complete pedigrees.

Although our initial focus was on exposure-induced meiotic recombination effects, we observed unexpected malformations of the vas deferens in two of the three CD-1 founder males. Accordingly, we evaluated the vas deferens of all descendant males and noted an increase in both the incidence and severity of defects with subsequent generations of exposure. Specifically, in addition to the abnormal kinking of the vas deferens observed in two F0 founders, an even more severe aberration that we termed ‘collapsed’ emerged in EE F1 males and increased in incidence in EEE F2 males (Fig 2). In addition, a new and severe phenotype, testis fibrosis, emerged in the third generation.

To assess the effect of successive generations of exposure on the incidence of vas deferens malformations (i.e., the proportion of males with either kinked or collapsed phenotypes), we compared F1 and F2 males with multiple generations of exposure (EE and EEE) to those with only one (E0 and E00). Among F1 males, the incidence of abnormalities was significantly increased in EE by comparison with E0 males (48.0% (12/25) vs. 10.0% (3/30), respectively; Χ^2^ = 8.1, p < 0.01; Fig 2D). A similar comparison of F2 males (EEE and E00) demonstrated an even stronger exposure effect (90.0% (36/40) vs. 11.4% (4/35), respectively; Χ^2^ = 43.2, p < 0.0001; Fig 2D). As a further test of the effect of successive generations of exposure, we compared F1 (EE) and F2 (EEE) males and found a significant increase in the incidence of defects in F2 males (Χ^2^ = 12.0, p < 0.001; Fig 2D).

The severity of reproductive tract abnormalities also increased with successive generations of exposure (Fig 2D and 2E). In the F1 generation, 28.0% (7/25) of EE but none (0/30) of the E0 males exhibited the more severe collapsed vas deferens phenotype (Fig 2D). In the F2 generation, the incidence of this severe abnormality was 47.5% (19/40) in EEE, but only 5.7% (2/35) in E00 grandsons (Fig 2D). Importantly, although the collapsed phenotype was evident in all three families, it was most pronounced in families 1 and 3 (Fig 2E, S1 Fig and S2 Fig). Notably, the family with the lowest incidence of the collapsed phenotype, family 2, was derived from the only founder with a normal vas deferens. Family 1 appeared most affected and, by comparison with family 2_,_ the collapsed phenotype occurred in 50.0% (4/8) vs.11.1% (1/9) of EE males (not significant), and 88.9% (8/9) vs. 25.0% (4/16) of EEE grandsons, respectively (Χ^2^ = 7.0, p < 0.01; Fig 2E). Intriguingly, in family 1 two E00 grandsons (18.2%) also exhibited the collapsed phenotype, providing the only examples of the severe phenotype among E00 males.

**Fig 3:**
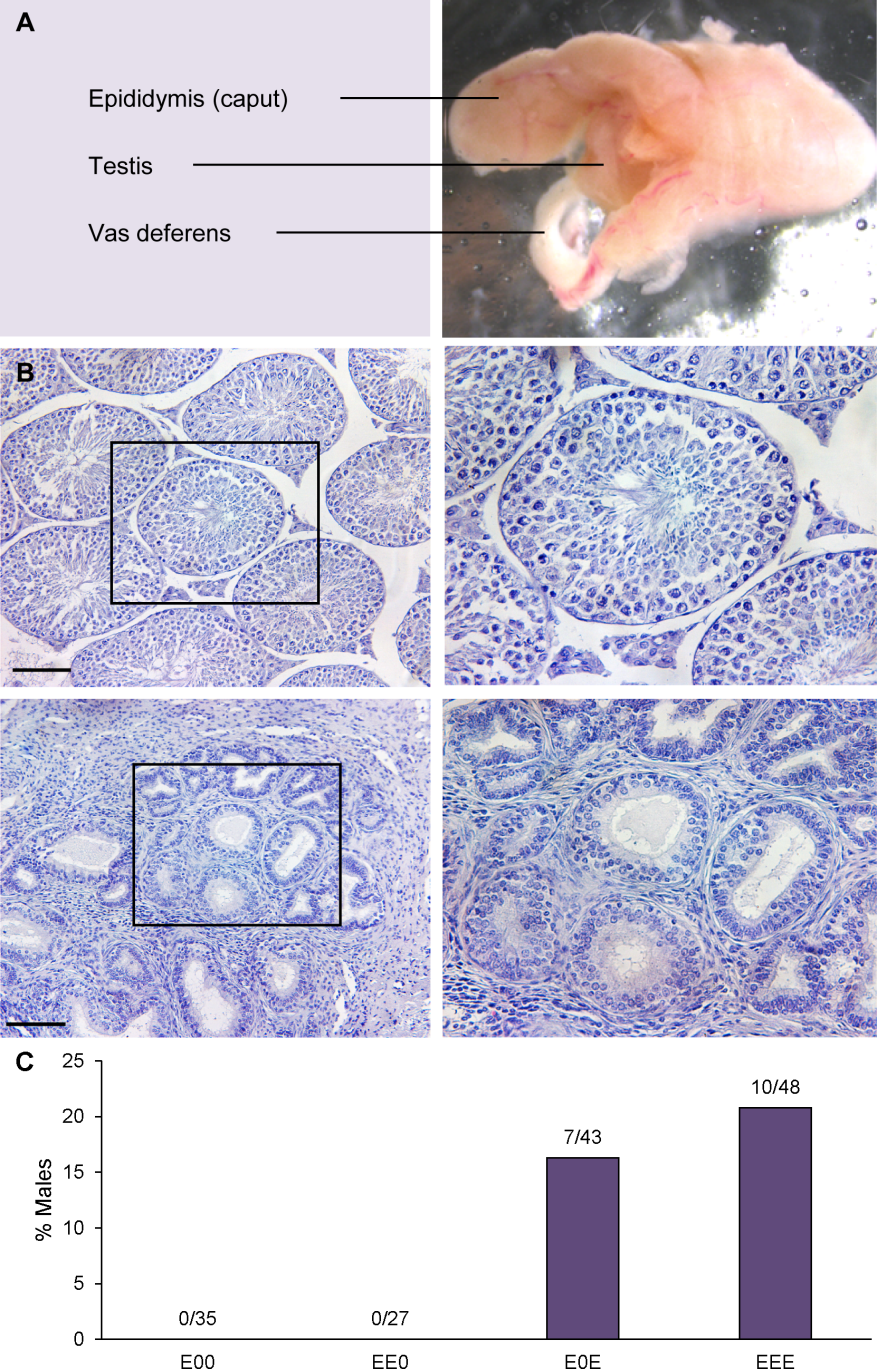
Fibrotic testis phenotype emerges after multiple generations of estrogenic exposure. (A) fibrotic testis from EEE male showing fusion of the epididymis, vas deferens, and testis. (B) Histological sections of control (top left) and fibrotic testis from EEE male (bottom left; scale bars denote 100 μm); black boxes indicate seminiferous tubules shown in high magnification images in right panels. By comparison with normal testis, fibrotic testis exhibits complete spermatogenic failure, with expansion of interstitial tissue and shrunken seminiferous tubules containing unhealthy Sertoli cells and spermatogonia. (C) Incidence of testicular fibrosis among third-generation males; number above each bar indicates number of males with fibrotic testes out of total scored.

Unexpected abnormalities were not confined to the ductal system, as a new, severe testis phenotype emerged in the third generation. Fibrotic testes, frequently characterized by fusion of the testis and reproductive tract (Fig 3A) was observed in a minority of F2 generation males in each exposure family (Fig 2E). As shown in Fig 3B, histological analysis of testes from affected males revealed prominent cysts (not evident in this image), apparent expansion of interstitial tissue, and atrophied seminiferous tubules devoid of active spermatogenesis. The phenotype was confined to F2 males but included individuals receiving either two or three generations of exposure, and in 14/17 cases both testes were affected. Among EEE males, the frequency was similar across families, with 20.0% (2/10), 21.1% (4/19), and 21.1% (4/19) affected for families 1, 2, and 3, respectively (Fig 2E). Unlike the vas deferens phenotype, the fibrotic testis phenotype was not obviously related to paternal phenotype, indeed 16.3% (7/43) of F2 males with an interrupted generation of exposure (i.e. E0E, but not EE0) exhibited fibrosis (Fig 3C, S1 Fig).

### Meiotic effects worsen with successive generations of exposure

In addition to eliciting more severe reproductive tract aberrations, multiple generations of estrogenic exposure exacerbated the meiotic recombination phenotype that was the original focus of our analysis. As in our previous studies [37], we analyzed recombination in pachytene stage spermatocytes by counting MLH1 foci in preparations immunostained with antibodies to both SYCP3 (a component of the synaptonemal complex or SC) and MLH1, a mismatch repair protein that localizes to the majority of meiotic crossovers [43]. In our previous studies, the MLH1 mean for placebo treated males was 24.6 ± 0.3 and both BPA and EE exposure induced a significant decrease (i.e., 1–2.5 foci, depending upon the exposure) [37]. Thus, the means of F0 founder males (23.7 ± 0.3, 22.7 ± 0.4, and 22.1 ± 0.3 for family 1, 2, and 3, respectively) fell within the expected range for exposed males. To compare recombination levels across generations and among different categories of F1 and F2 males, mean MLH1 counts were derived by pooling cells from males of the same generation and exposure category.

**Fig 4:**
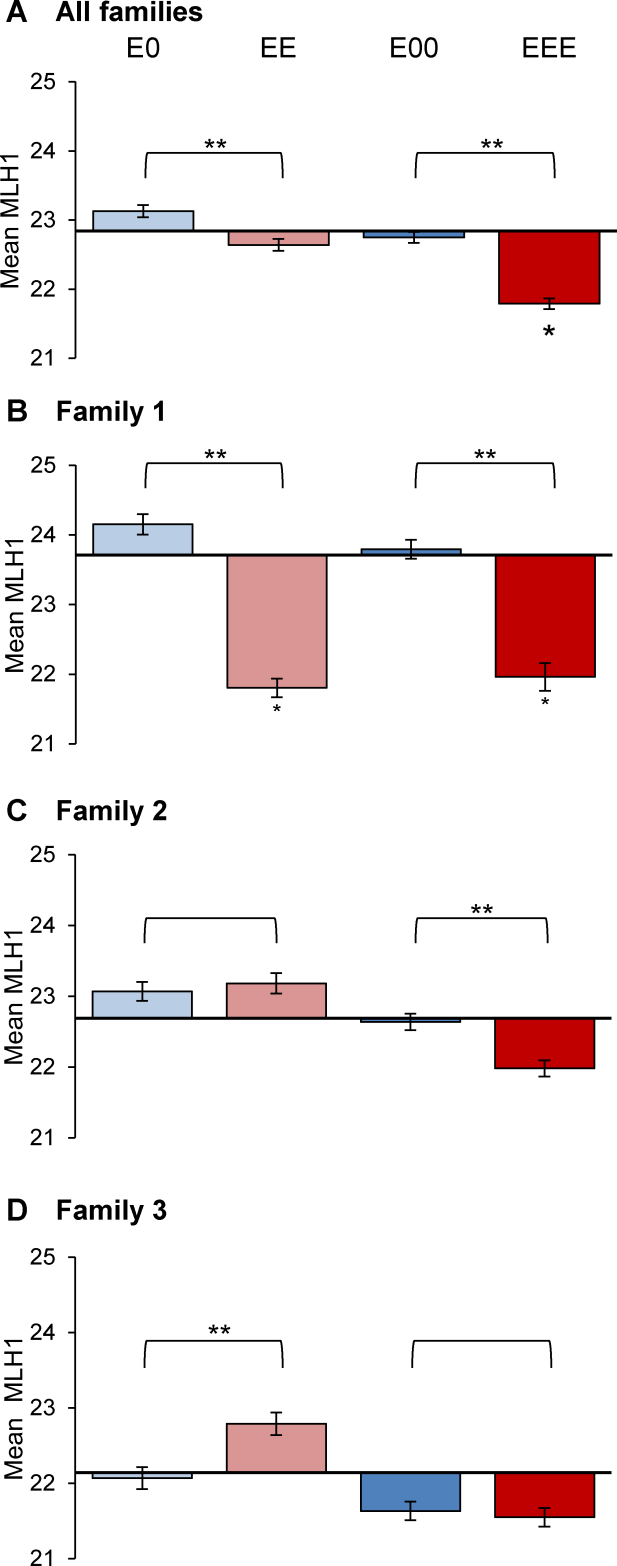
Meiotic recombination levels decrease with successive generations of exposure. (A) Pooled data from exposed families. X-axis represents MLH1 mean of 3 F0 founder males (25–30 pachytene cells/male) and bars show mean ± SEM for E0 and EE F1 sons and E00 and EEE F2 grandsons. Bar color denotes individual exposure (red for exposed, blue for placebo) with increased intensity for successive generations of exposure or placebo. Each group represents data from 25–30 pachytene stage cells/male for 22–23 males. (B-D) Individual data for families 1, 2, and 3 (B, C, and D, respectively); each group represents 4–12 males. Groups were compared by one-way ANOVA; single asterisk denotes significant difference by comparison with founder and double asterisk denotes significance between indicated groups as determined by Tukey-Kramer post-hoc test (p < 0.05).

To assess the effect of successive generations of exposure, we used one-way ANOVA to compare mean MLH1 counts in exposed F0 males with those in F1 and F2 males exposed for two or three generations (Fig 4A; F = 29.4, p < 0.0001). Significant differences between groups were determined by a Tukey-Kramer post-hoc test. By comparison with F0 founders (22.8 ± 0.2) we found a small but nonsignificant decrease in mean MLH1 counts in EE sons (22.7 ± 0.1), but a significant reduction in EEE grandsons (21.8 ± 0.1; p < 0.05). In addition, MLH1 means were lower in F1 EE (22.7 ± 0.1) than E0 sons (23.1 ± 0.1, p < 0.05). Similarly, the mean was significantly lower in EEE by comparison with E00 F2 males (21.8 ± 0.1 and 22.8 ± 0.1, respectively; p < 0.05).

Because family 1 exhibited the strongest effect, we assessed each family individually to determine if trends were consistent across families (Fig 4B–4D). In family 1, MLH1 means were significantly lower in both F1 EE sons (21.8 ± 0.1) and F2 EEE grandsons (22.0 ± 0.2) by comparison with the founder mean (23.7 ± 0.3; p < 0.05; Fig 4B). For families 2 and 3, reductions were evident in F2 EEE males, but the differences were not statistically significant (Fig 4C and 4D). Thus, all families exhibited the same trend; differences among them prompted us to consider a paternal effect on recombination.

### Recombination exhibits a strong paternal effect

As observed for vas deferens abnormalities, the recombination phenotype of offspring appeared to be influenced by paternal phenotype. Specifically, the extent to which the phenotype worsened with successive generations of exposure not only varied among families, but also among the offspring of males within a family, with a more pronounced effect in sons of males with higher mean MLH1 counts. For example, the founder of family 1 had the highest mean MLH1 level (23.7 ± 0.3), and his seven F1 sons (EE) all had lower mean values (ranging from 21.0 ± 0.3 to 23.4 ± 0.3; Fig 4B, S3 Fig). In contrast, in the other two families where founder MLH1 means were lower (22.7 ± 0.4, and 22.1 ± 0.3 for family 2 and 3, respectively), means in F1 EE sons (23.2 ± 0.1 and 22.8 ± 0.2, respectively) were not significantly different from the F0 founder mean (Fig 4C and 4D).

**Fig 5:**
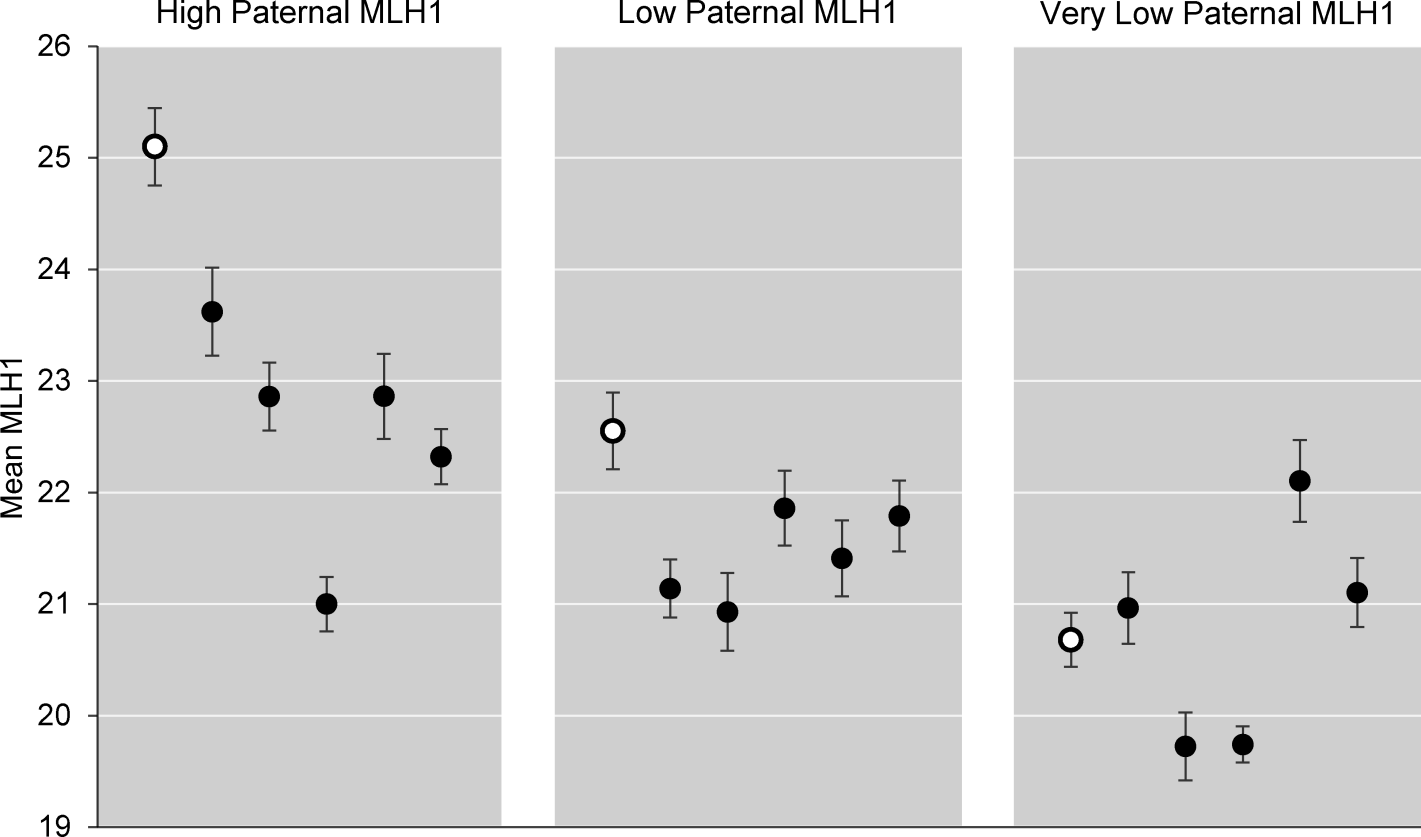
Paternal phenotype affects meiotic recombination levels. Mean MLH1 ± SEM for F1 EE fathers (open circle) and their F2 EEE sons (closed circles). Each point represents the MLH1 mean ± SEM for 25–30 pachytene cells from a single male. Left and center panels show offspring data from family 2 for two F1 EE fathers with different means: 25.1 ± 0.4 (high paternal MLH1), and 22.6 ± 0.3 (low paternal MLH1). Right panel shows offspring data for EE father with a very low mean, 20.7 ± 0.2, from family 3. Fathers and offspring were compared by one-tailed t-test; for high paternal MLH1, p < 0.0001; for low paternal MLH1 p < 0.01.

A comparison of the F2 sons of F1 EE fathers provided further evidence of this paternal effect. For example, the two F1 EE males in family 2 that were mated to produce F2 EEE males had very different MLH1 means (25.1 ± 0.4 and 22.6 ± 0.3). Although the five F2 EEE offspring of each male had lower mean MLH1 counts than their fathers (23.0 ± 0.2, t = 4.6, p < 0.0001, and 21.4 ± 0.2, t = 3.1, p < 0.01 respectively; Fig 5), the means and ranges of the two groups of males were remarkably different. Importantly, the magnitude of the reduction was greater in F2 sons of the F1 male with the high MLH1 count. Similar paternal effects were observed among the offspring in all three families (S3 Fig); however, the impact of the paternal phenotype on the response to exposure was most pronounced in family 3, where the MLH1 mean of one F1 male was particularly low (20.7 ± 0.2). The mean for the F2 EEE sons of this male (20.1 ± 0.2) did not differ significantly from the F1 EE father, making this the only group of F2 EEE males that did not demonstrate an additional reduction in recombination levels by comparison with their father (Fig 5).

### MLH1 null SCs increase in frequency with successive generations of exposure

**Fig 6:**
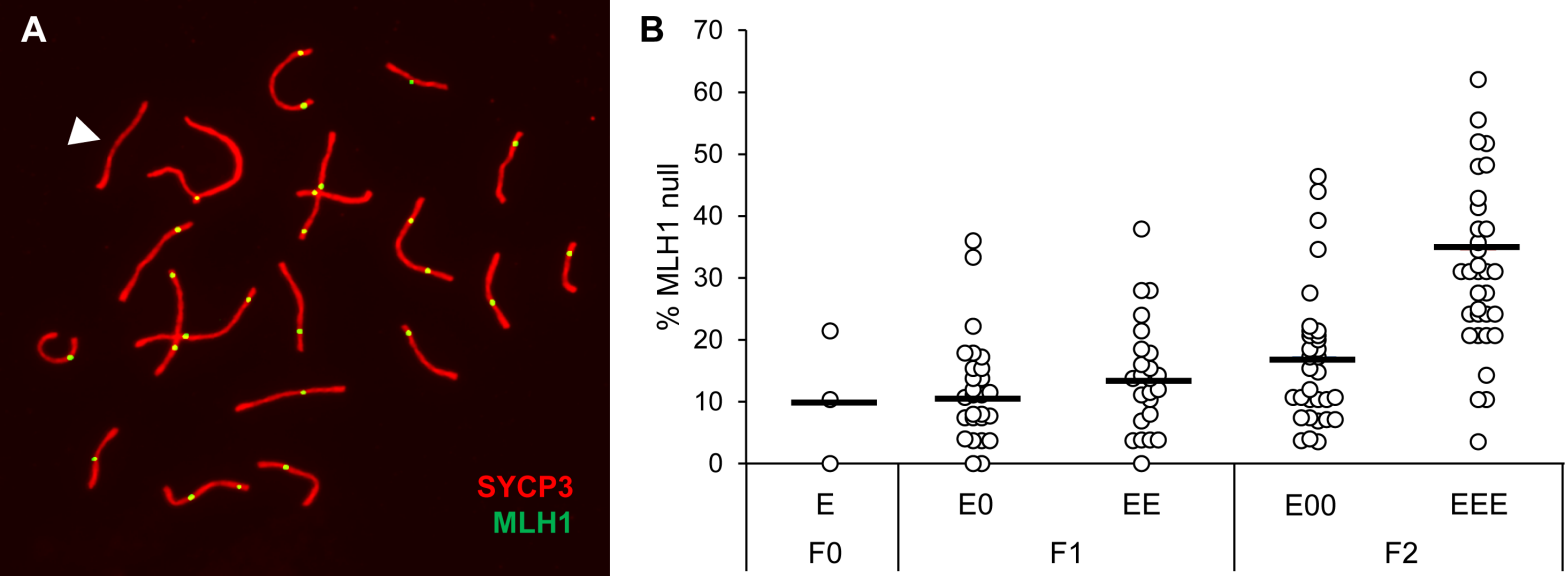
Cells with SCs lacking an MLH1 focus increase in frequency with successive generations of exposure. (A) Example of a pachytene spermatocyte immunostained with antibodies to SYCP3 (red) and MLH1 (green), and showing an SC lacking an MLH1 focus (white arrowhead). (B) Frequency of MLH1 null SCs in 3 founder males, 28 E0 and 24 EE F1 sons, and 32 E00 and 25 EEE F2 grandsons (25–30 cells analyzed per male).

The variability induced by the use of outbred males, coupled with the male reproductive tract abnormalities we encountered, confounded the use of standard measurements of impaired male fertility. Thus, we elected to directly measure meiotic impairment by scoring cells with lethal defects, i.e., the frequency of pachytene stage cells containing one or more SCs lacking an MLH1 focus (MLH1 null SCs). As expected, exposure-induced reductions in meiotic recombination resulted in an increase in MLH1 null SCs (Fig 6A). A comparison of males exposed each generation (i.e. E, EE, and EEE) showed a significant increase in the incidence of these cells over three successive generations. Specifically, MLH1 null SCs were observed in 10.8% (9/83) of cells from F0 founder males, 14.2% (91/643) of cells from F1 EE sons, and 34.9% (248/710) of cells from F2 EEE grandsons (Χ^2^ = 87.9, p < 0.0001; Fig 6B). Although the difference in levels of SCs that fail to form an exchange between founders and EE sons was not significant, levels in EEE grandsons were significantly higher by comparison with both founders (Χ^2^ = 18.6, p < 0.0001) and EE fathers (Χ^2^ = 76.5, p < 0.0001). This trend held among father-son comparisons within individual families (S4 Fig).

The MLH1 null phenotype also provided a means of assessing the transgenerational persistence of meiotic effects. A comparison of levels in E, E0, and E00 males not only did not reveal a decrease in the frequency of cells with MLH1 negative SCs levels in subsequent unexposed generations but provided evidence of a slight increase across generations (Χ^2^ = 11.2, p < 0.01; Fig 6B). However, the effect was only statistically significant in the pooled data and a definitive trend was not observed across all families (S4 Fig). Thus, although these data are consistent with transgenerational persistence of the phenotype, clearly additional analyses are warranted.

### Both ancestral and individual exposure influences the male recombination phenotype

As detailed above, our data from exposure families provide evidence of increases in both the severity of reproductive tract aberrations and meiotic effects with successive generations of exposure. Our breeding scheme generated three different types of two-generation exposure males (F1 EE males, and F2 E0E and EE0 males), and these males provide a means of separating ‘ancestral’ and direct or ‘individual’ exposure effects. To assess the effect of individual exposure, we compared EE0 and E0E F2 males. Ancestral exposure is common to both, but only E0E males received direct exposure as neonates. A comparison of pooled data for these F2 males suggests stronger effects as a result of individual exposure. Vas deferens defects were more common in E0E males, with abnormal phenotypes in 87.2% (34/39) males vs. 15.4% (4/26) in the EE0 males (Χ^2^ = 30.2, p < 0.0001; S5A Fig). These defects were also more severe in E0E males, with collapsed phenotypes in 38.5% (15/39) of males vs. 3.8% (1/26) of EE0 males (Χ^2^ = 8.3, p < 0.01; S5A Fig). Similarly, a stronger reduction in MLH1 counts was evident in E0E males, with means of 22.1 ± 0.07 for E0E and 22.8 ± 0.10 for EE0 (Tukey-Kramer post-hoc, p < 0.05; S5B Fig). Further, the proportion of cells with MLH1 null SCs was also higher: 26.4% (198/749 cells) for E0E vs 18.2% (115/633 cells) for EE0 (Χ^2^ = 12.9, p < 0.001; S5C Fig). These trends held when we examined individual families with one exception: in family 3, E0E males showed a lower, although not significant, decrease in MLH1 null SCs by comparison with EE0 males (16.6% (42/253) and 20.1% (56/279), respectively; S6 Fig).

## Discussion

Growing evidence suggests that developmental exposure to EDCs may exert effects that span multiple generations (e.g., [19,21,22,24,25,44–48]), but little attention has focused on an equally important question with obvious human relevance—the effect of successive generations of exposure. In this respect, our study is unique since, rather than exposing one generation and assessing the persistence of effects in subsequent generations, we exposed multiple generations and compared the severity of effects across generations. Further, in contrast to trans- and multigenerational studies that have used pooled data to assess effects in each generation, we assessed differences among individual families, focusing on relationships between exposure effects evident in fathers, sons, and grandsons.

Most reports of transgenerational effects have utilized *in utero* exposures that can disrupt fetal development as well as epigenetic reprogramming of the germline. Instead, we attempted to target the developing germline by exposing male mice postnatally during the period thought to coincide with the establishment of the spermatogonial stem cell (SSC) pool [49]. Effects elicited in the male reproductive tract demonstrate that our exposure also affected somatic differentiation of the male reproductive tract but, importantly, these unexpected effects lend further support to the conclusions of our meiotic studies.

The major finding from our study is that, in male mice, estrogenic exposure spanning several generations exacerbates reproductive abnormalities induced by exposure. Our exposure paradigm (Fig 1) allowed us to identify both ancestral and paternal effects on the incidence and intensity of exposure phenotypes. First, ancestral exposure influenced the magnitude of the effect, since exposed grandsons–even those sired by nonexposed fathers—exhibited the most severe reproductive aberrations. Second, paternal phenotype strongly affected the magnitude of the meiotic effect in exposed offspring. Taken together, our data add a concerning new dimension to the estrogen hypothesis [8]. Specifically, our findings suggest that neonatal estrogenic exposure can affect both the reproductive tract and sperm production in exposed males, and exposure effects are exacerbated by exposure spanning multiple generations. Because estrogenic chemicals have become both increasingly common and ubiquitous environmental contaminants in developed countries, the implications for humans are serious. Indeed, it is possible effects are already apparent, with population-based studies from the U.S., Europe, Japan, and China reporting reductions in sperm counts/quality [2,3,5,6,50–53] and male fertility (reviewed in [7]) within a span of several decades.

### Successive generations of estrogenic exposure exacerbate male reproductive effects

We identified three phenotypes that increased in severity with successive generations of neonatal estrogenic exposure. Two of the effects, malformations of the vas deferens and altered levels of meiotic recombination, were evident in first generation exposure males (F0) but were exacerbated by successive generations of exposure. The third and most severe phenotype, fibrotic testes, was observed only in third-generation (F2) males.

The vas deferens, which is normally a straight tubule, exhibited kinking along its entire length in two of the three founder males. This phenotype not only was more pronounced in exposed descendants, but became markedly more severe (i.e., ‘collapsed’) after two generations of successive exposure (28% of EE males; Fig 2) and was the predominant phenotype in EEE males (47.5%; Fig 2).

Altered Hox gene expression is known to affect the developing male reproductive tract, and similar abnormalities of the vas deferens—described as partial homeotic transformation of the vas deferens into an epididymis—have been reported in *Hoxa10* and *Hoxa11* mutant adults [54–56]. In our studies, however, and in postnatal DES exposure studies where similar vas deferens effects were observed [57–59], fetal development of the male reproductive tract would be unaffected. Thus, the abnormalities induced by postnatal exposure must result from delayed or impaired differentiation. Because the proximal-to-distal coiling of the epididymis concludes around the time of birth [60] our findings, in conjunction with those from DES exposed males, suggest that neonatal exposure to estrogens prevents cessation of coiling in the male reproductive tract. A critical role of androgens in the perinatal elongation and coiling of the differentiating Wolffian ducts/epididymis has been suggested previously [60,61], and DES-induced reproductive tract defects have been suggested to result from disruption of the neonatal estrogen-androgen balance [58,59]. Our data, however, add a new level of complexity regarding sensitivity to exposure: Although ethinyl estradiol and DES have similar IC50 and relative binding affinities for estrogen receptors [62], the effects we observed among EEE grandsons were more severe than with DES-induced abnormalities [57–59] despite the fact that the level of estrogenic exposure was markedly lower (18.3 ng ethinyl estradiol vs. 60 μg DES). Thus, our data suggest that sensitivity to exposure is increased with successive generations of exposure.

The emergence of a new phenotype, fibrotic testes exhibiting complete spermatogenic failure, in the F2 generation further underscores the cumulative consequences of multiple generations of exposure. Because this phenotype only emerged in the last generation and was not evident until the males were killed for analysis, we can only speculate on the genesis of this abnormality. However, given the high frequency of orchitis encountered when we attempted to initiate studies using inbred C3H males, it seems likely that exposure may interfere with the formation of the blood-testis barrier. In addition to being common (20.8% and 16.3% of EEE and E0E males, respectively; Fig 3C), the fibrotic phenotype is particularly interesting because a similar phenotype has been reported in CD-1 males following prenatal exposure to high doses (100 ng/g) of DES [63]. Because we utilized a considerably lower dose of ethinyl estradiol (0.25 ng/g), the appearance of this phenotype in third-generation males supports the hypothesis that exposure sensitivity is heightened by multiple generations of exposure.

### Meiotic effects intensify with successive generations of estrogenic exposure

The phenotype that was our original focus of study, reduced levels of meiotic recombination in exposed males, also was exacerbated by successive generations of exposure. To assess effects on the germline, we analyzed the mean number of MLH1 foci in pachytene spermatocytes from 12-week-old males. MLH1 is a DNA mismatch repair protein that localizes in pachytene cells to future sites of crossovers and thus serves as a useful surrogate for meiotic recombination. Recombination levels in mice have been well characterized and are influenced by genetic background, with marked differences among inbred strains [64–66]. Growing evidence, however, suggests that the testicular environment also exerts strong effects. For example, there are at least two different age effects on recombination levels in male mice. MLH1 counts are strikingly lower in the first wave of spermatocytes in the juvenile testis, but rapidly rise to adult levels within a matter of days [67]. These recombination changes coincide with the maturation of the testosterone producing Leydig cells of the testis. A second age effect, a slight, but significant increase in MLH1 levels, is observed in inbred male mice 1 year of age and older [67]. We postulate that these age-related changes in meiotic recombination reflect epigenetic changes to the germline induced by changes in the testis environment. Importantly, the meiotic effects induced by neonatal estrogen exposure that we observed in the present study support this conclusion: The decrease in MLH1 foci over multiple generations is presumably a downstream effect of exposure-induced changes to the developing SSC population. Although exposure may affect the somatic lineages of the testis, previous SSC transplantation experiments demonstrate that the meiotic recombination phenotype results from changes induced in SSCs [37], suggesting that the SSC itself is responsive to changing endogenous hormone signals or exposure to estrogenic EDCs. As our studies demonstrate, effects induced in developing SSCs by brief postnatal exposure have profound effects, including permanent alterations that not only persist in all meiotic descendants of SSCs in exposed males, but also across multiple generations. Although it is well established that recombination hotspots are epigenetically regulated, the mechanism linking epigenetic changes in the SSC to altered meiotic recombination levels in spermatocytes many cell divisions downstream remains unknown. Our current studies are focused on identifying and understanding the changes in the SSC epigenome that are induced by estrogenic exposure, thereby gleaning new insight to the control of meiotic recombination in mammals as well as to the risks posed to our reproductive health by EDCs common in our daily environment.

MLH1 counts provide a sensitive and quantitative means of assessing the effects of estrogenic exposure [37] and, for the purpose of the present study, a means of comparing effects across generations. Mean MLH1 counts in three generation exposure lineage F2 males (EEE) were not only significantly lower than levels in their grandfathers (F0 founder males), but also by comparison with single generation exposure F2 males (E00). Although the recombination changes may appear subtle, from the standpoint of successful spermatogenesis, they are substantial. The mouse genome consists of twenty pairs of chromosomes, and a physical connection (chiasma), between each pair is essential for normal segregation of homologous chromosomes at the first meiotic division. Because chiasmata are established at sites of meiotic recombination, an exposure-induced reduction in MLH1 counts should increase the incidence of SCs lacking an MLH1 focus, an expectation borne out in our previous studies [37]. Our current results not only confirm this finding, but demonstrate a worsening of the effect with increasing generations of exposure. Indeed, the 35% incidence of cells with MLH1 null SCs observed in EEE grandsons (Fig 6B) is particularly concerning. Failure to form a crossover increases the incidence of univalents at metaphase I, and these cells are effectively eliminated from the spermatocyte pool through the robust actions of the spindle assembly checkpoint (SAC; [41,42]), a finding confirmed in our initial studies [37]. Thus, the net effect of recombination changes induced by successive generations of estrogenic exposure would be reduced levels of sperm production. Because sperm counts are variable among outbred males, and the high incidence of reproductive tract abnormalities would confound simple fertility measurements, we did not attempt to quantitate the exposure effect on male fertility. Based on the MLH1 null SC levels observed in F0 founders (10.8%) and F1 EE males (14.2%), however, we suspect that the effects of exposure in the first two generations are too subtle to elicit significant effects on fertility. In contrast, although we did not breed F2 males the high incidence of testis fibrosis in this generation suggests a marked increase in infertility with successive generations of exposure.

### Paternal phenotype affects the severity of exposure effects in descendants

The incidence and severity of meiotic and reproductive tract defects varied among the three exposure families in our study. CD-1 is an outbred strain and, to obtain successive generations, males were mated with unexposed females. Thus, phenotypic variation is expected. The variability among males, however, allowed us to discern an effect of paternal phenotype on the severity of meiotic effects in subsequent generations. Specifically, a larger reduction in mean MLH1 counts was evident among exposed sons of fathers with high recombination levels by comparison with sons of fathers with very low recombination levels (Fig 5). In essence, our data raise the possibility of a maximal effect of estrogenic exposure on meiotic recombination that, once reached, cannot be further exacerbated. Thus, in future studies it will be important to assess effects of exposure spanning more than three generations.

### Ancestral and individual exposures exacerbate reproductive defects

Our data also provide evidence of the persistence of ancestral exposure effects. Cumulative effects of multiple generations of exposure provide strong evidence that a combination of both ancestral and individual exposures elicits the most pronounced effects, as F2 males with both (e.g., E0E grandsons) exhibited more frequent and severe phenotypes than did males without individual exposure (EE0 grandsons). Further, it is notable that testicular fibrosis was observed only in males with both grandpaternal and individual exposures as neonates (i.e., E0E and EEE males), providing further evidence that the combination of an ancestral and individual exposure was both necessary and sufficient to elicit the most severe reproductive effects.

### Summary and implications for human health

Increasing evidence that developmental exposure to endocrine disrupting chemicals can elicit phenotypic effects that are transgenerationally inherited has sparked interest in exposure-induced epigenetic changes (reviewed in [68,69]). Associated changes in DNA methylation [70], histone modifications [71], and small RNAs [47,72] have been reported, but causative links between these alterations and transgenerational disease phenotypes are equivocal. Few studies have focused on changes to the germline (e.g., [26,28]) or traced the transmission of specific epimutations across generations (e.g., [27,71]). The complex multigenerational exposure paradigm that we used allowed for the detection of exposure effects directly in germ cells and made it possible to quantitatively compare effects in descendant males. Thus, in addition to assessing trans- and multigenerational effects, our study represents a logical and important next step in exposure studies—assessing the effects of multiple generations of exposure. Because both exposure-induced meiotic and reproductive tract defects increased in frequency and severity with successive generations of exposure, our data provide evidence that persistent exposure increases male reproductive tract sensitivity. Given the dramatic increase in both the number and complexity of environmental chemical contaminants during the past several decades, our findings have obvious human relevance. Indeed, evidence of global reductions in sperm production [1,3,6,50–53,73] suggest that similar effects may already be manifest in human populations, and underscore the importance of understanding the levels and types of exposures in human populations.

## Materials and methods

### Ethics statement

Our studies using mice were approved by the Institutional Animal Care and Use Committee at Washington State University under protocol number 03745, and all animals used in these studies were treated humanely and in accordance with the American Association for Accreditation of Laboratory Animal Care guidelines (institution animal welfare assurance number A3485-01). Animals were killed for analysis using inhalation of carbon dioxide, and death was confirmed using cervical dislocation.

### Animals

Outbred CD-1 mice (Harlan Laboratories) were housed in polysulfone cages on ventilated racks (Allentown Inc., Jag 57 micro isolator model) in a pathogen-free facility. Cages contained Sanichip 7090A bedding (Harlan Laboratories) and a nestlet (Ancare) for enrichment. Drinking water and food (Purina Lab Diet, 5K52) were provided ad libitum.

All experiments were approved by the International Animal Care and Use Committee (IACUC) at Washington State University, which is fully accredited by the American Association for Accreditation of Laboratory Animal Care.

### Exposures

All males were treated from 1–12 days postpartum (dpp) with either 0.25 ng/g/day ethinyl estradiol (Sigma-Aldrich, E4876) or equal volume ethanol/corn oil placebo. Ethinyl estradiol was dissolved in 100% ethanol and diluted in tocopherol-stripped corn oil (MP Biomedicals) and administered orally by pipette. Doses were calculated based on mean pup weight (g) for this strain. The 0.25 ng/g ethinyl estradiol dose was chosen because it was used as a positive control in our previous studies and elicited a strong meiotic effect [37]. The dose used is roughly equivalent to that in contraceptive pills (15–30 μg).

Three estrogen-exposed males served as the F0 founders of three exposure families. At 6 wks. of age, these founder males were paired with unrelated CD-1 females to produce second-generation (F1) males. Each founder produced four litters; two were treated with ethinyl estradiol and two with placebo. At sexual maturation, one randomly-selected male from each litter was mated to produce four litters of third-generation (F2) males, two treated with ethinyl estradiol and two placebo-treated. This paradigm yielded two groups of F1 males with either one (E0) or two (EE) generations of exposure (n = 30 and 25 mice, respectively), and four groups of F2 males having: 1) a single ancestral exposure (E00; n = 35); 2) an ancestral and paternal exposure (EE0; n = 27); 3) an ancestral and individual exposure (E0E; n = 43); or 4) three successive generations of exposure (EEE; n = 48; Fig 1). Males of all generations were killed at 12 wks. of age and their testes and reproductive tracts removed for analysis. Similarly, males from unexposed lineages (n = 27) were treated with placebo from 1–12 dpp, killed at 12 wks. of age, and their testes and reproductive tracts were analyzed as a control group.

### Reproductive tract analysis

Vas deferens and epididymides were dissected and images captured using a Leica DFC295 camera on a Leica dissection microscope. The morphology of the vas deferens was assigned a numerical score of 1 (normal), 2 (kinked), or 3 (collapsed) by three independent observers who were blinded with respect to exposure status.

### Immunohistochemistry

Testes exhibiting signs of fibrosis were fixed in Bouins solution, embedded in paraffin, and sectioned. Sections were deparaffinized, rehydrated, and stained with hematoxylin.

### Spermatocyte preparations and immunostaining

Spermatocyte preparations were made according to the method developed by Peters [74]. Slides were incubated overnight in a humid chamber and washed with 0.4% Photo-flo 200 solution (Kodak Professional). Immunofluorescence staining of slides was performed as described previously [37]. Slides were simultaneously stained with MLH1 primary antibody (Calbiochem, PC56, at 1:60) and SYCP3 primary antibody (Santa Cruz biotechnology, sc-74569, at 1:300), and counterstained with Alexa Fluor 488-conjugated AffiniPure Donkey Anti-Rabbit (AFDAR) secondary antibody (Jackson Immunoresearch Laboratories, Inc., 711-545-152, at 1:60) and Cy3-conjuagted AffiniPure Donkey Anti-Mouse (CDAM) secondary antibody (Jackson Immunoresearch Laboratories, Inc., 715-165-150, at 1:1000).

### MLH1 analysis

Cells were imaged using a Zeiss Axio Imager epifluorescence microscope. MLH1-FITC, SYCP3-TRITC, and DAPI were imaged sequentially, adjusted using Zeiss Axiovision software, and the number of MLH1 foci in composite images of MLH1 and SYCP3 counted by two independent observers who were blinded with regard to exposure status. 25–30 pachytene spermatocytes were scored per animal; minor counting discrepancies were resolved but cells with major scoring discrepancies, poor staining, or synaptic defects were excluded.

### Statistical analyses

Among-group differences in mean MLH1 foci counts were analyzed by one-way ANOVA. For statistically significant differences (p < 0.05), a Tukey-Kramer post-hoc test was performed to infer which groups differed. Comparisons of mean MLH1 foci counts between F1 fathers and their F2 sons were analyzed by one-tailed t-test. Chi-square analyses were used to determine significance in the proportion of vas deferens aberrations and of cells containing SCs lacking an MLH1 focus.

## Supporting information

S1 Fig Pedigrees of three exposure families. For each family, blue lines of descent indicate placebo and red lines estrogen treatment. Vas deferens phenotype of each male is denoted by square color: normal (white), kinked (light blue), and collapsed (dark blue), with black squares denoting fibrotic testes. Only one EEE and EE0 lineage were obtained in family 1 because the second F1 EE father died in cage.
(TIF)

S2 Fig Frequency of vas deferens abnormalities in individual families. Frequency of normal (light blue), kinked (medium blue), and collapsed (dark blue) phenotypes; each family consists of 9–12 E0 and 8–9 EE F1 sons, and 11–12 E00 and 9–16 EEE F2 grandsons. Comparisons of incidence of abnormal phenotypes: family 1: for E0 and EE, Χ^2^ = 8.0 (p < 0.05); for E00 and EEE, Χ^2^ = 11.5 (p < 0.01); family 2: for E00 and EEE, Χ^2^ = 17.1 (p < 0.0001); for EE and EEE, Χ^2^ = 7.7 (p < 0.01); family 3: for E00 and EEE, Χ^2^ = 16.7 (p < 0.0001).
(TIF)

S3 Fig Paternal phenotype affects meiotic recombination levels. Mean MLH1 ± SEM for F0 founders (black) and their F1 EE sons (light red) for families 1, 2, and 3 (left panels). Arrows denote F1 males used to sire F2 offspring. Center and right panels show mean MLH1 ± SEM for F1 EE fathers (light red) and their F2 EEE sons (dark red). Each point represents a single male (25–30 pachytene cells). Fathers and offspring were compared by one-tailed t-test. For family 1: F0 founder v. F1 EE sons (t = 5.3, p < 0.0001); F1 EE father v. F2 EEE sons right panel (t = 3.4, p < 0.001). For family 2: F1 EE father v. F2 EEE sons: center panel (t = 4.6, p < 0.0001); right panel (t = 3.1, p < 0.01). For family 3: F1 EE father v. F2 EEE sons: center panel (t = 5.5, p < 0.0001).
(TIF)

S4 Fig Comparison of MLH1 null frequency in fathers and sons. Frequency of cells with MLH1 negative SCs in F1 fathers and their F2 sons for each family. Bar color denotes individual exposure (red for exposed, blue for placebo), increased intensity denotes additional generation of exposure or placebo. Each EE or E0 group represents of 7–11 males (25–30 cells per male); all other groups consist of 7–14 F2 males (25–30 cells per male).
(TIF)

S5 Fig Phenotypic severity is influenced by ancestral and individual exposures. Comparison of 25 F1 EE males and 26 EE0 and 39 E0E F2 males. (A) Frequency of kinked (light blue) and collapsed (dark blue) vas deferens morphology. Incidence of abnormal phenotypes was significantly higher in males with both ancestral and individual exposures (E0E): For EE and E0E, Χ^2^ = 9.7 (p < 0.01); for EE0 and E0E Χ^2^ = 30.2 (p < 0.0001). Severity of vas defects was also higher in E0E by comparison with EE0 males, Χ^2^ = 8.3 (p < 0.01) (B) Mean MLH1 ± SEM; 25–30 cells/male. X-axis represents F0 founder mean. Asterisk denotes significant difference as determined using a Tukey-Kramer post-hoc test (p < 0.05). (C) Frequency of MLH1-null SCs. For EE and E0E, Χ^2^ = 31.0 (p < 0.0001), and for EE0 and E0E Χ^2^ = 12.9 (p < 0.001).
(TIF)

S6 Fig Exposure/phenotype by family. Comparison of F1 EE males (n = 8, 9, and 8) and EE0 (n = 4, 12, and 10) and E0E (n = 16, 13, and 10) F2 males from families 1, 2, and 3, respectively. (A) Frequency of kinked (light blue) and collapsed (dark blue) vas deferens morphology. For family 2: EE and E0E, Χ^2^ = 8.8 p < 0.01; EE0 and E0E, Χ^2^ = 21.2, p < 0.0001. For family 3: EE and E0E, Χ^2^ = 4.3 p < 0.05; EE0 and E0E, Χ^2^ = 6.5, p < 0.05. (B) Mean MLH1 ± SEM; 25–30 cells/male. X-axis represents founder mean. Asterisk denotes significant difference as determined using a Tukey-Kramer post-hoc test (p < 0.05). (C) Frequency of MLH1-null SCs. For family 1: EE and E0E, Χ^2^ = 7.2 (p < 0.01); EE0 and E0E, Χ^2^ = 12.5 (p < 0.001). For family 2: EE and E0E, Χ^2^ = 27.1 (p < 0.0001); EE0 and E0E, Χ^2^ = 11.1 (p < 0.001). For family 3: EE and EE0, Χ^2^ = 4.9 (p < 0.05).
(TIF)
