# Correction: Germline and reproductive tract effects intensify in male mice with successive generations of estrogenic exposure

**DOI:** 10.1371/journal.pgen.1006980

**Published:** 2017-08-30

**Authors:** Tegan S. Horan, Alyssa Marre, Terry Hassold, Crystal Lawson, Patricia A. Hunt

Following the publication of this article [1], the authors were made aware that the aberrant seminiferous tubules shown in Fig 3B look like invasive cribriform growth of the caput epididymal epithelium into the testis. The legend for Fig 3 has been updated to include this information, and a new supporting figure (S7 Fig) can be viewed below. Staining with GNCA failed to detect germ cells in these tubules, and subsequent clusterin staining (S7 Fig) is consistent with the presence of epididymal principal cells. Because disruption of estrogen signaling has been reported to elicit cribriform growth and granulomas in the epididymis and aberrant growth into the efferent ducts (reviewed in [2]), the rare tubules observed in this otherwise fibrotic testis appear to be the result of extreme disruption of estrogen-regulation of epididymal physiology.

**Fig 3:**
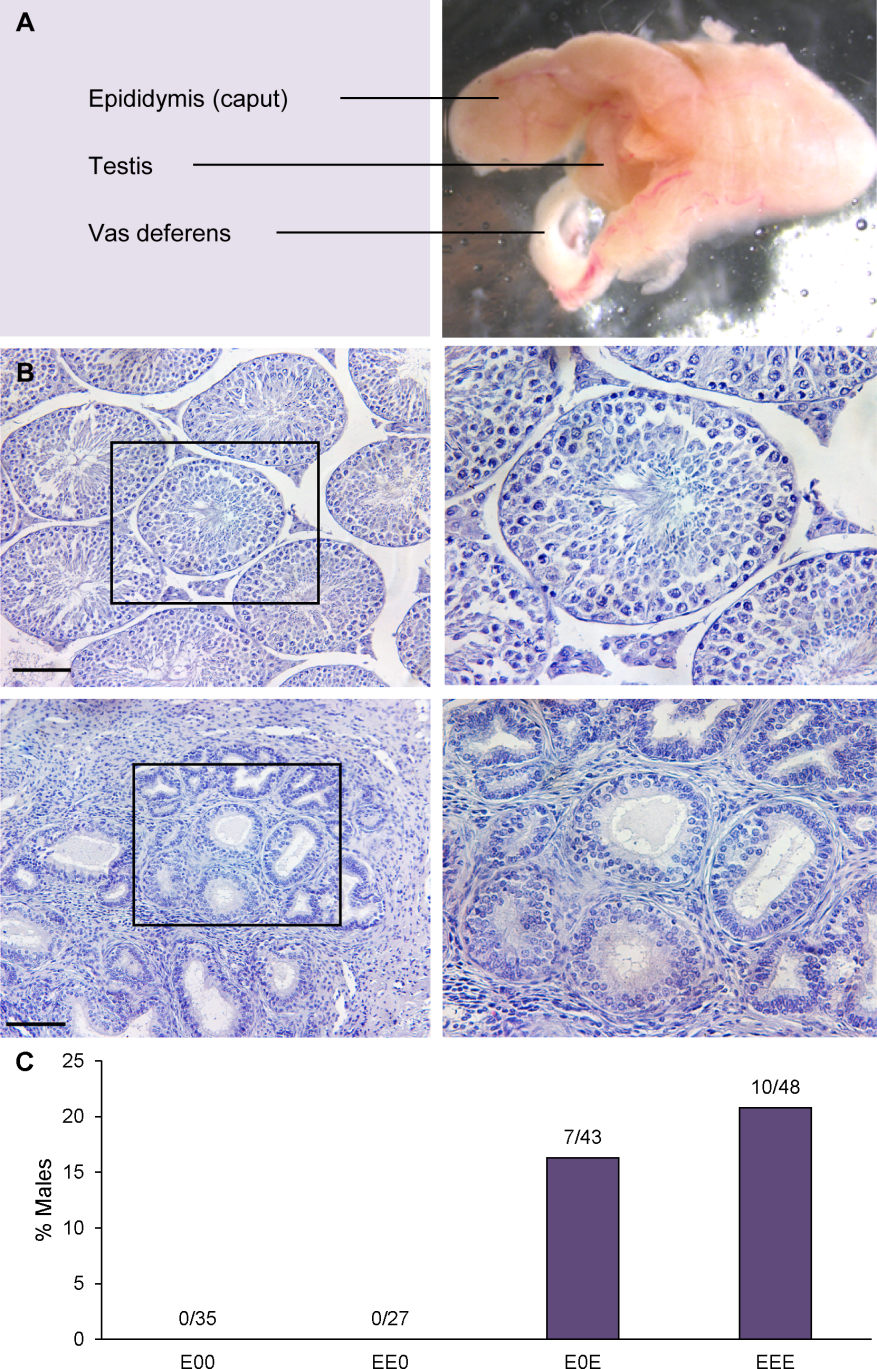
Fibrotic testis phenotype emerges after multiple generations of estrogenic exposure. (A) fibrotic testis from EEE male showing fusion of the epididymis, vas deferens, and testis. (B) Histological sections of control (top left) and fibrotic testis from EEE male (bottom left; scale bars denote 100 μm); black boxes indicate seminiferous tubules (control) and invasive cribriform growth of caput epididymal epithelium into the testis (EEE) shown in high magnification images in right panels. By comparison with normal testis, fibrotic testis exhibits loss of both seminiferous tubules and germ cells, with expansion of interstitial tissue. (C) Incidence of testicular fibrosis among third-generation males; number above each bar indicates number of males with fibrotic testes out of total scored.

## Supporting information

S7 Fig Aberrant growth of caput epididymal epithelium into fibrotic testis. Histological section of fibrotic testis from EEE male stained with an antibody to clusterin (brown), a marker of epididymal principal cells. Scale bar denotes 50 µm.
(TIF)
