# Supplementary figures and images for: Germline and reproductive tract effects intensify in male mice with successive generations of estrogenic exposure
Source: PLoS Genet. 2017 Jul 20;13(7):e1006885. doi: 10.1371/journal.pgen.1006885 (PMC5519010; doi:10.1371/journal.pgen.1006885)

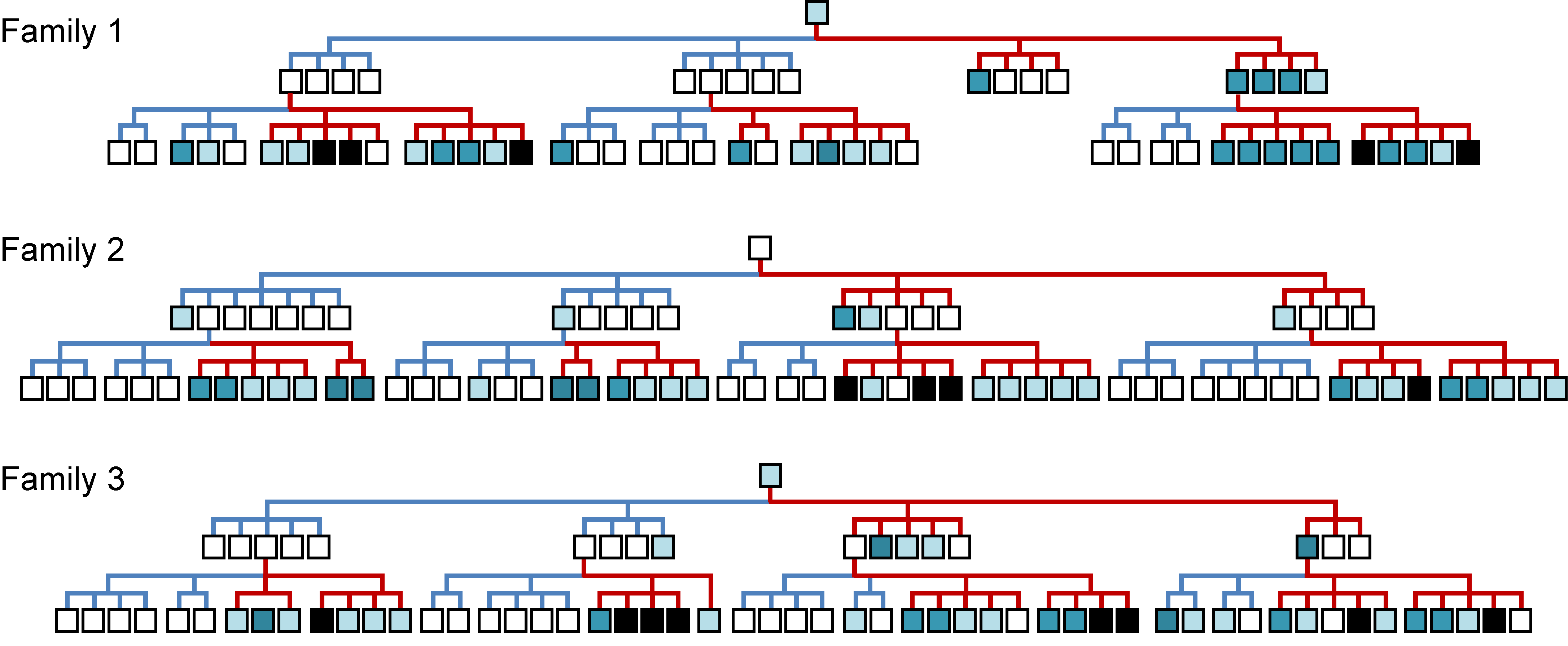

Supplement: S1 Fig — For each family, blue lines of descent indicate placebo and red lines estrogen treatment. Vas deferens phenotype of each male is denoted by square color: normal (white), kinked (light blue), and collapsed (dark blue), with black squares denoting fibrotic testes. Only one EEE and EE0 lineage were obtained in family 1 because the second F1 EE father died in cage. (TIF) [file pgen.1006885.s001.tif]

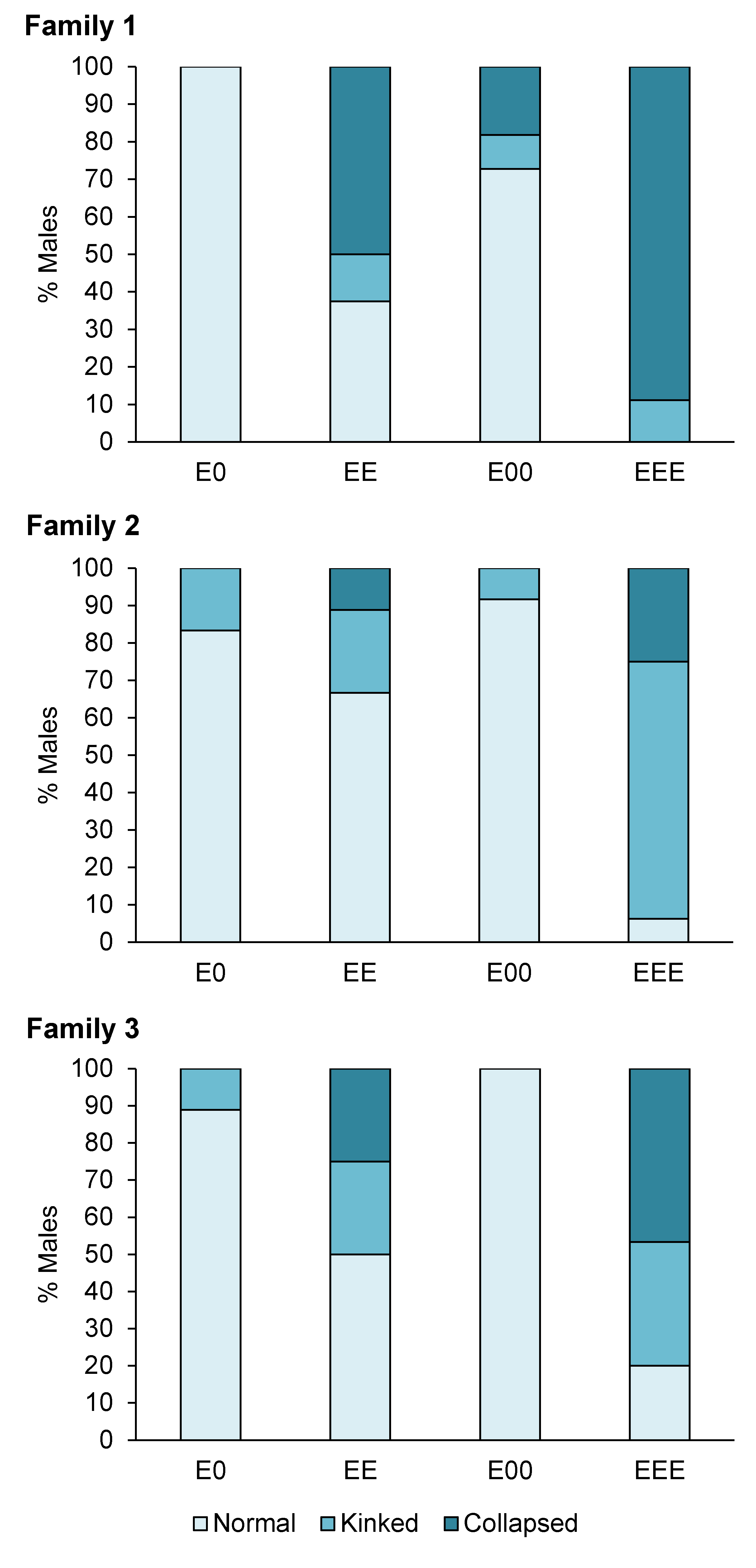

Supplement: S2 Fig — Frequency of normal (light blue), kinked (medium blue), and collapsed (dark blue) phenotypes; each family consists of 9–12 E0 and 8–9 EE F1 sons, and 11–12 E00 and 9–16 EEE F2 grandsons. Comparisons of incidence of abnormal phenotypes: family 1: for E0 and EE, Χ2 = 8.0 (p < 0.05); for E00 and EEE, Χ2 = 11.5 (p < 0.01); family 2: for E00 and EEE, Χ2 = 17.1 (p < 0.0001); for EE and EEE, Χ2 = 7.7 (p < 0.01); family 3: for E00 and EEE, Χ2 = 16.7 (p < 0.0001). (TIF) [file pgen.1006885.s002.tif]

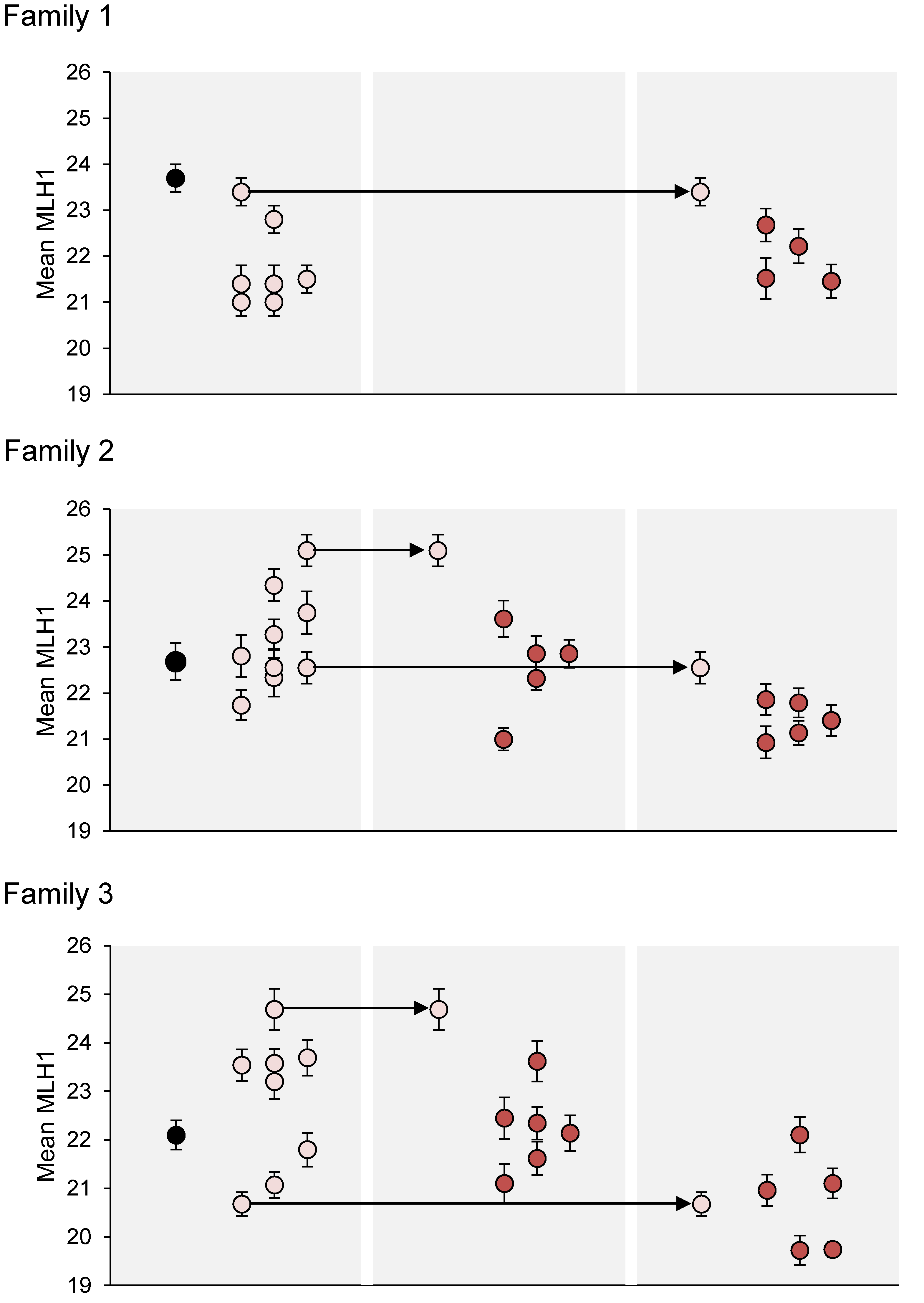

Supplement: S3 Fig — Mean MLH1 ± SEM for F0 founders (black) and their F1 EE sons (light red) for families 1, 2, and 3 (left panels). Arrows denote F1 males used to sire F2 offspring. Center and right panels show mean MLH1 ± SEM for F1 EE fathers (light red) and their F2 EEE sons (dark red). Each point represents a single male (25–30 pachytene cells). Fathers and offspring were compared by one-tailed t-test. For family 1: F0 founder v. F1 EE sons (t = 5.3, p < 0.0001); F1 EE father v. F2 EEE sons right panel (t = 3.4, p < 0.001). For family 2: F1 EE father v. F2 EEE sons: center panel (t = 4.6, p < 0.0001); right panel (t = 3.1, p < 0.01). For family 3: F1 EE father v. F2 EEE sons: center panel (t = 5.5, p < 0.0001). (TIF) [file pgen.1006885.s003.tif]

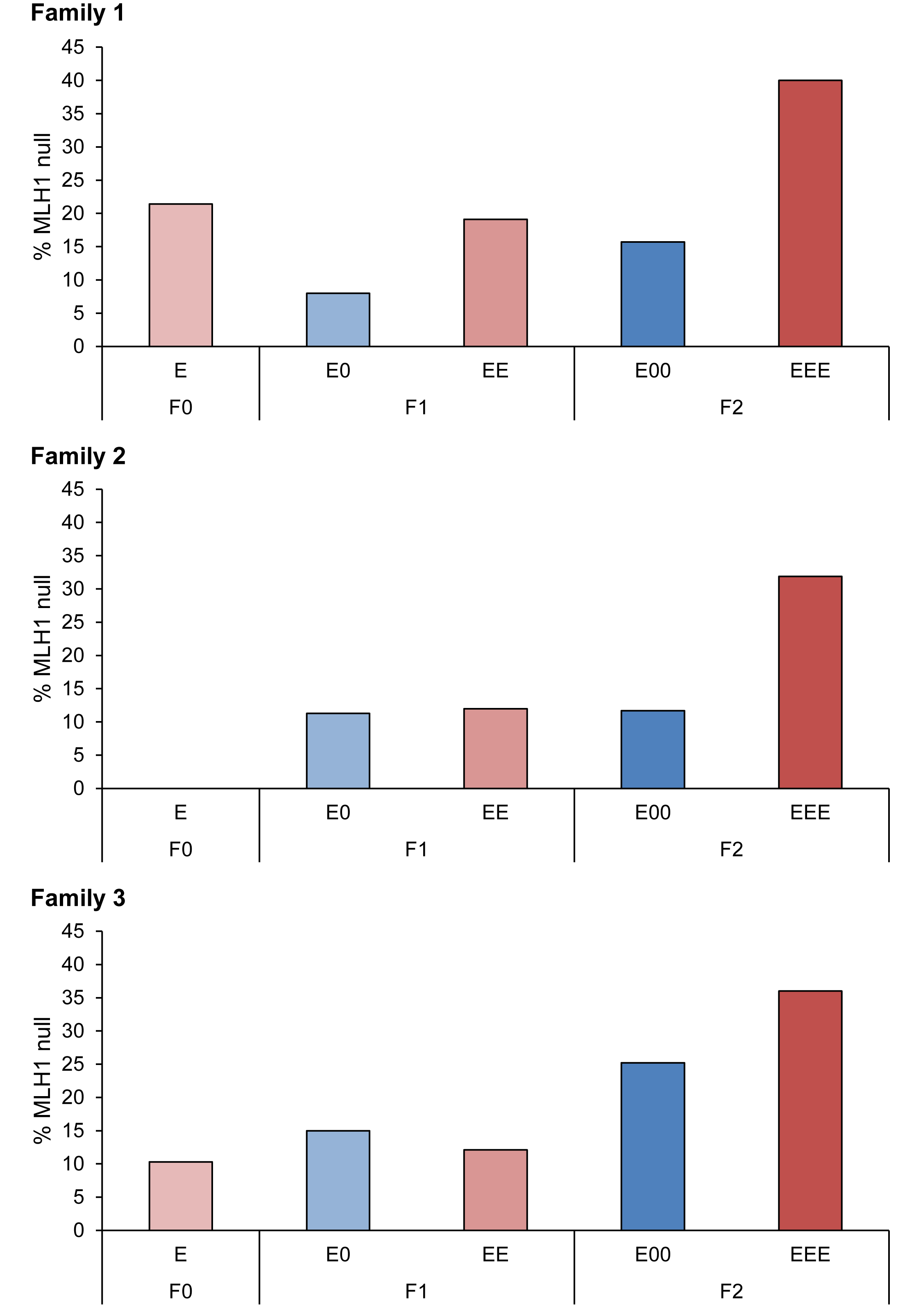

Supplement: S4 Fig — Frequency of cells with MLH1 negative SCs in F1 fathers and their F2 sons for each family. Bar color denotes individual exposure (red for exposed, blue for placebo), increased intensity denotes additional generation of exposure or placebo. Each EE or E0 group represents of 7–11 males (25–30 cells per male); all other groups consist of 7–14 F2 males (25–30 cells per male). (TIF) [file pgen.1006885.s004.tif]

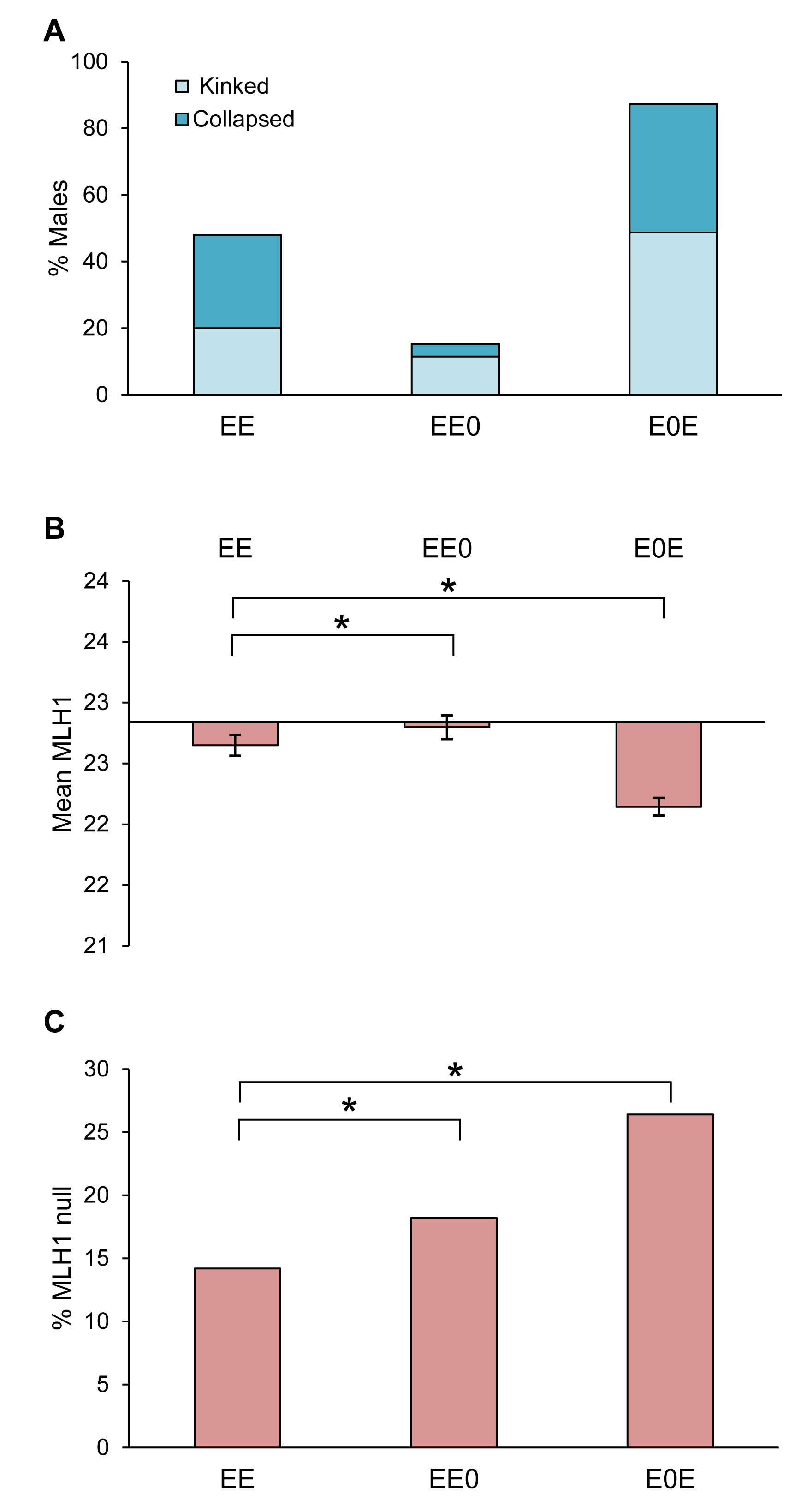

Supplement: S5 Fig — Comparison of 25 F1 EE males and 26 EE0 and 39 E0E F2 males. (A) Frequency of kinked (light blue) and collapsed (dark blue) vas deferens morphology. Incidence of abnormal phenotypes was significantly higher in males with both ancestral and individual exposures (E0E): For EE and E0E, Χ2 = 9.7 (p < 0.01); for EE0 and E0E Χ2 = 30.2 (p < 0.0001). Severity of vas defects was also higher in E0E by comparison with EE0 males, Χ2 = 8.3 (p < 0.01) (B) Mean MLH1 ± SEM; 25–30 cells/male. X-axis represents F0 founder mean. Asterisk denotes significant difference as determined using a Tukey-Kramer post-hoc test (p < 0.05). (C) Frequency of MLH1-null SCs. For EE and E0E, Χ2 = 31.0 (p < 0.0001), and for EE0 and E0E Χ2 = 12.9 (p < 0.001). (TIF) [file pgen.1006885.s005.tif]

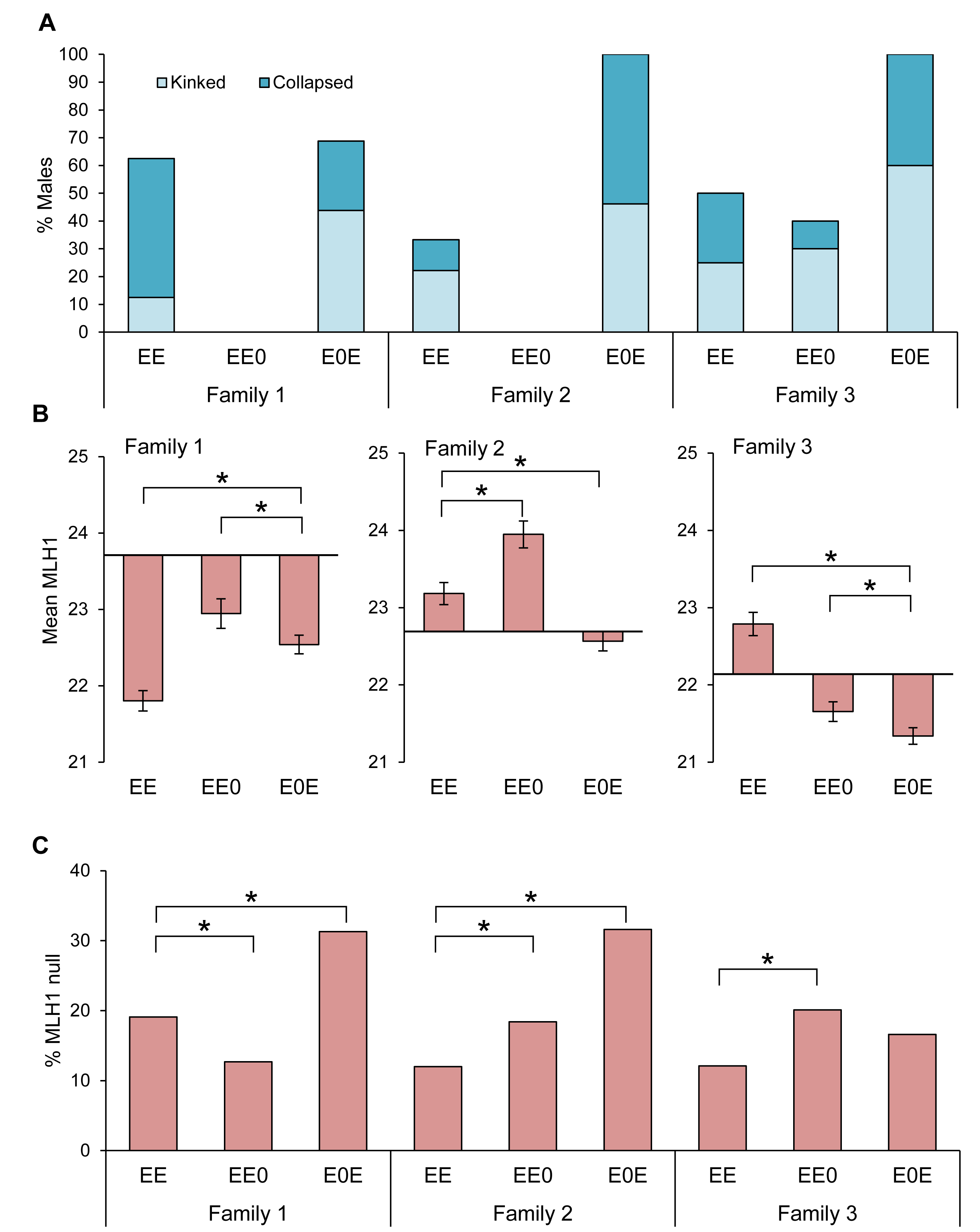

Supplement: S6 Fig — Comparison of F1 EE males (n = 8, 9, and 8) and EE0 (n = 4, 12, and 10) and E0E (n = 16, 13, and 10) F2 males from families 1, 2, and 3, respectively. (A) Frequency of kinked (light blue) and collapsed (dark blue) vas deferens morphology. For family 2: EE and E0E, Χ2 = 8.8 p < 0.01; EE0 and E0E, Χ2 = 21.2, p < 0.0001. For family 3: EE and E0E, Χ2 = 4.3 p < 0.05; EE0 and E0E, Χ2 = 6.5, p < 0.05. (B) Mean MLH1 ± SEM; 25–30 cells/male. X-axis represents founder mean. Asterisk denotes significant difference as determined using a Tukey-Kramer post-hoc test (p < 0.05). (C) Frequency of MLH1-null SCs. For family 1: EE and E0E, Χ2 = 7.2 (p < 0.01); EE0 and E0E, Χ2 = 12.5 (p < 0.001). For family 2: EE and E0E, Χ2 = 27.1 (p < 0.0001); EE0 and E0E, Χ2 = 11.1 (p < 0.001). For family 3: EE and EE0, Χ2 = 4.9 (p < 0.05). (TIF) [file pgen.1006885.s006.tif]
