# Supplementary figures and images for: Correction: Germline and reproductive tract effects intensify in male mice with successive generations of estrogenic exposure
Source: PLoS Genet. 2017 Aug 30;13(8):e1006980. doi: 10.1371/journal.pgen.1006980 (PMC5576655; doi:10.1371/journal.pgen.1006980)

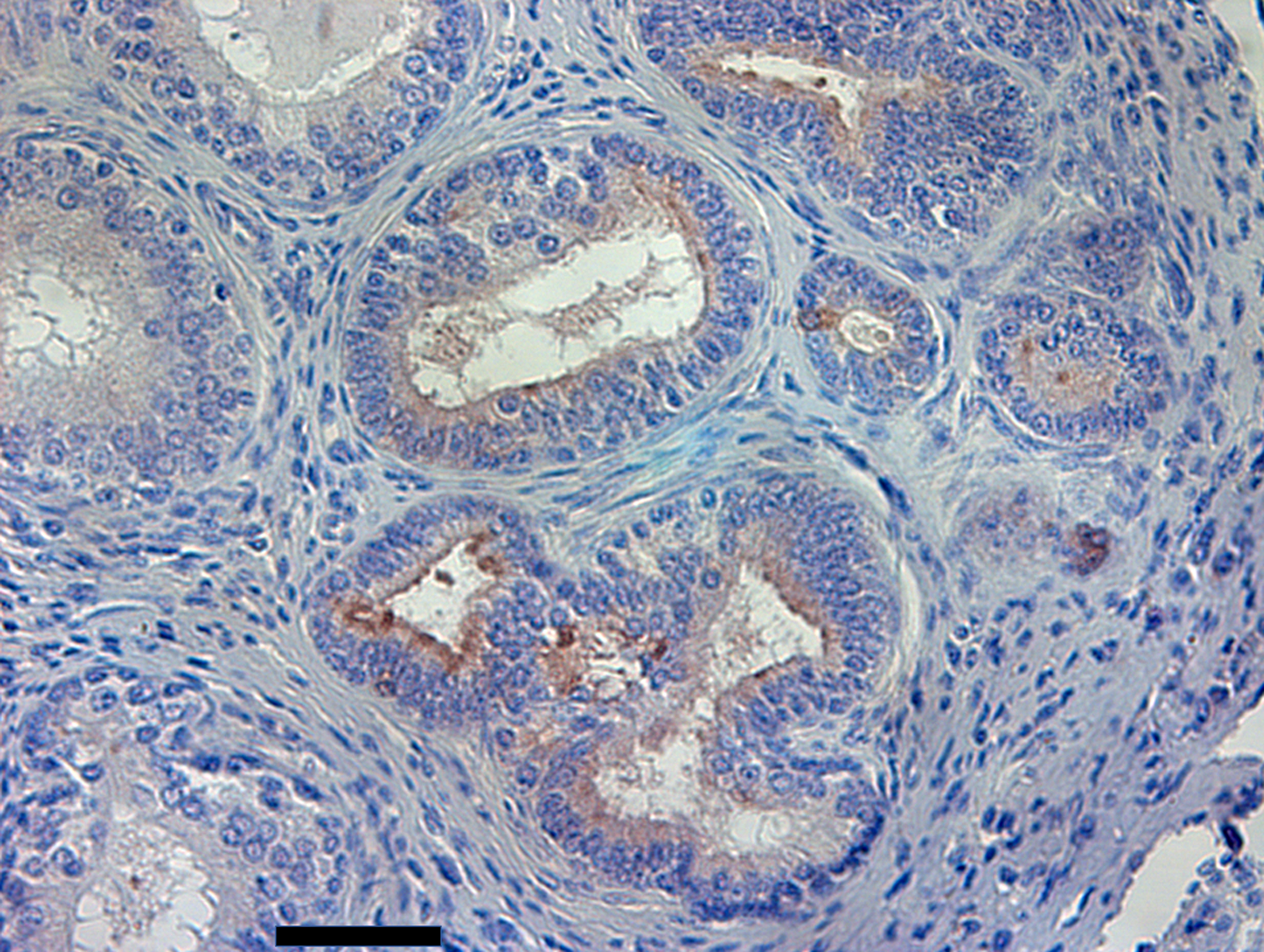

Supplement: S7 Fig — Histological section of fibrotic testis from EEE male stained with an antibody to clusterin (brown), a marker of epididymal principal cells. Scale bar denotes 50 µm. (TIF) [file pgen.1006980.s001.tif]
